# Right Atrial Mass With Pulmonary Embolism and Tricuspid Dysfunction: Features Favoring Right-Sided Infective Endocarditis

**DOI:** 10.7759/cureus.102645

**Published:** 2026-01-30

**Authors:** Lucia P Schroeder, Rosie Kumar, Maha Fathali, Nicolas Thor

**Affiliations:** 1 Internal Medicine, Arrowhead Regional Medical Center, Colton, USA; 2 Internal Medicine, California University of Science and Medicine, Colton, USA

**Keywords:** cardiac vegetation, endocarditis, right atrial mass, right-sided endocarditis, tricuspid regurgitation, vegetation

## Abstract

Right-sided infective endocarditis (RSIE) represents a minority of infective endocarditis cases but carries substantial morbidity due to embolic and hemodynamic complications, particularly in patients with intravenous drug use. We describe a 45-year-old man with intravenous drug use who presented in septic shock complicated by disseminated intravascular coagulation and peripheral gangrene. Blood cultures grew *Streptococcus agalactiae*. Multimodal cardiac imaging revealed a large, mobile right atrial mass prolapsing through the tricuspid valve, resulting in severe tricuspid regurgitation and associated pulmonary embolism. Despite diagnostic uncertainty between thrombus and infective vegetation, cumulative clinical, microbiologic, and imaging features supported a working diagnosis of RSIE. Given the prohibitive surgical risk, percutaneous aspiration using the AngioVac system (AngioDynamics, Latham, NY, USA) was pursued following multidisciplinary evaluation. This case highlights a practical escalation framework for percutaneous intervention in critically ill patients with right-sided cardiac masses when diagnostic certainty is limited but embolic risk and hemodynamic compromise are substantial.

## Introduction

Right-sided cardiac masses pose a significant diagnostic and therapeutic challenge, with a differential diagnosis that includes thrombus, benign or malignant tumors, and infective endocarditis (IE) [1,2]. Right-sided IE (RSIE) accounts for approximately 5-10% of IE cases and is most commonly associated with intravenous drug use, indwelling vascular access, or intracardiac devices [1,3]. Although less common than left-sided IE, RSIE is associated with considerable morbidity due to embolic phenomena, severe tricuspid regurgitation, and right ventricular dysfunction.

Large right atrial (RA) masses are particularly uncommon and often present with overlapping imaging characteristics, making definitive differentiation between vegetation and thrombus challenging, especially in critically ill patients [2]. Transesophageal echocardiography (TEE) remains the cornerstone of evaluation, while multimodal imaging - including computed tomography (CT) - provides complementary information regarding mass morphology, attachment, embolic burden, and hemodynamic consequences. Management decisions frequently require multidisciplinary collaboration, particularly when surgical risk is prohibitive and tissue diagnosis cannot be obtained.

We report a complex case of septic shock complicated by a large RA mass causing severe tricuspid regurgitation and pulmonary embolism. This case underscores the diagnostic uncertainty inherent to right-sided cardiac masses and highlights the role of multimodal imaging and multidisciplinary decision-making in guiding escalation to percutaneous intervention.

## Case presentation

A 45-year-old man with a history of intravenous drug use, polysubstance use disorder, and chronic hepatitis C was transferred for higher-level care after presenting in septic shock with altered mental status and rapidly progressive peripheral skin changes. He was found unresponsive with new violaceous lesions involving the extremities and required vasopressor support and broad-spectrum intravenous antibiotics at the referring facility.

On arrival, the patient was tachycardic and toxic-appearing. Physical examination revealed diffuse purpura, symmetric peripheral gangrene of the hands and feet, and oozing from vascular access sites. Laboratory studies (Table 1) demonstrated marked leukocytosis, severe thrombocytopenia, acute kidney injury, transaminitis, hyperbilirubinemia, and coagulopathy consistent with disseminated intravascular coagulation (DIC). Blood cultures returned positive within 24 hours for Streptococcus agalactiae, prompting targeted antimicrobial therapy with intravenous beta-lactam antibiotics per infectious disease recommendations.

**Table 1:** Initial Laboratory Findings MCH: mean corpuscular hemoglobin, MCV: mean corpuscular volume, RDW: red cell distribution width, BUN: blood urea nitrogen, eGFR: estimated glomerular filtration rate, ALT: alanine aminotransferase, AST: aspartate aminotransferase, CK: creatine kinase, LDH: lactate dehydrogenase, INR: international normalised ratio, aPPT: activated partial thromboplastin time, DDU: D-dimer units

Parameter	Patient Value	Reference Range
WBC	54.2	4.5 - 11.1 x10^3 /uL
RBC	3.79	4.5 - 5.90 x10^6 /uL
Hemoglobin	10.8	13.0 - 17.0 x10 g/dL
Hematocrit	31	41 - 53%
MCV	83	80 - 100 fL
MCH	28.5	26 - 32 pg
RDW	14	11 - 15%
Platelets	31	120 - 360x10^3 /uL
Bands	19	<10%
Sodium	125	135 - 148 mmol/L
Potassium	4.6	3.5 - 5.5 mmol/L
Chloride	91	98 - 110 mmol/L
CO2	17	24- 35 mmol/L
BUN	109	8 - 20 mg/dL
Creatinine	2.54	0.50 - 1.50 mg/dL
eGFR	30.9	mL/min/1.73m^2
Glucose	101	65 - 125 mg/dL
Calcium	7.8	8.5 - 10.5 mg/dL
Phosphorus	8.5	2.4 - 4.4 mg/dL
Magnesium	2.9	1.6 - 2.3 mg/dL
Alkaline Phosphate	196	35 - 125 U/L
Albumin	2.5	3.5 - 4.9 g/dL
Total Protein	5.7	6 - 8 g/dL
AST	180	5 - 40 U/L
ALT	80	5 - 40 U/L
Total Bilirubin	8.5	0.0 -1.2 mg/dL
Total CK	1568	30 - 170 U/L
LDH	852	120 - 230 U/L
Troponin T	24.20	<=20.00 ng/L
Prothrombin time	26.8	11.8 - 14.2 seconds
INR	2.69	<=1.10
aPTT	40.5	25.4 - 36.8 seconds
Fibrinogen	197	174 - 482 mg/dL
D-dimer	5010	0 - 250 ng/ml DDU

**Figure 1:**
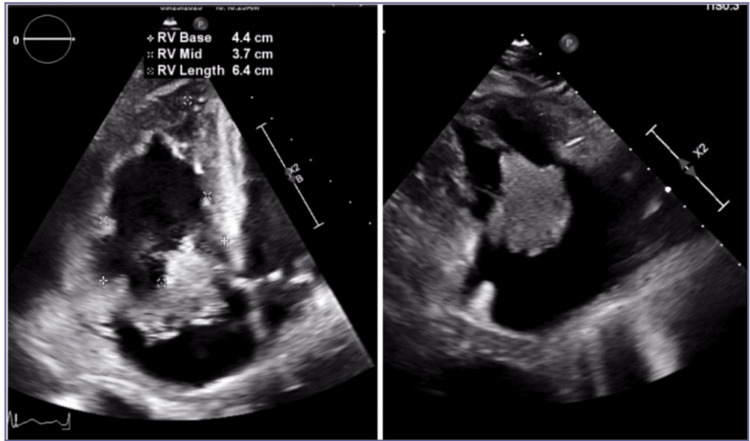
Transesophageal echocardiography demonstrating a 3 × 3 × 3 cm lobulated right atrial mass with a narrow pedunculated attachment just superior to the anterior tricuspid annulus. The mass prolapses through the tricuspid valve into the right ventricle during diastole, resulting in severe tricuspid regurgitation. Imaging features suggest a high likelihood of infective vegetation rather than thrombus, guiding multidisciplinary management decisions.

Bedside transthoracic echocardiography suggested a right-sided intracardiac mass. Following partial hemodynamic stabilization in the intensive care unit, TEE revealed a large (3 × 3 × 3 cm), lobulated, highly mobile RA mass with a narrow pedunculated attachment just superior to the anterior tricuspid annulus (Figure 1). The mass prolapsed into the right ventricle during diastole, resulting in severe tricuspid regurgitation, right ventricular dilation, and systolic dysfunction. These findings raised concern for an infective vegetation versus an organized thrombus.

**Figure 2:**
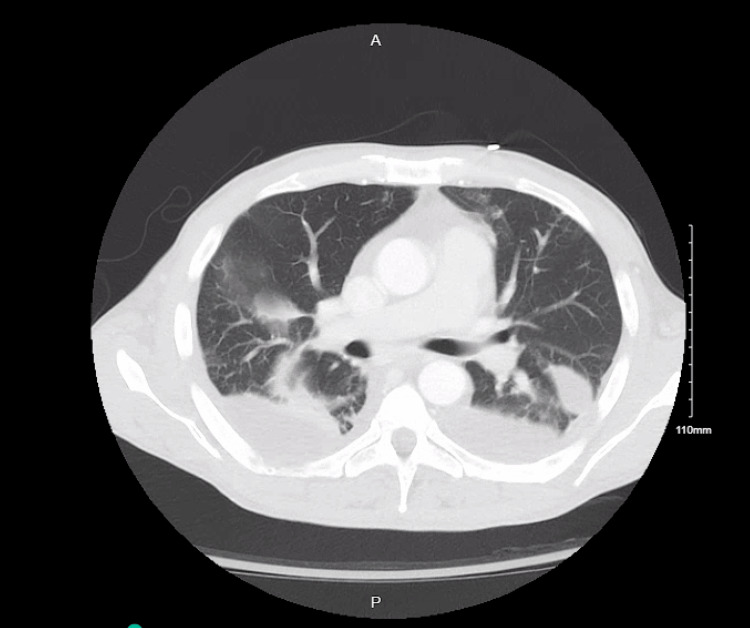
CT Pulmonary Angiogram CT pulmonary angiogram showing a right segmental pulmonary embolism, scattered bilateral cavitary pulmonary lesions suspicious for septic emboli, and bilateral loculated pleural effusions.

CT pulmonary angiography subsequently demonstrated a left segmental pulmonary embolism (Figure 2), presumed septic in the context of bacteremia and intracardiac mass. Systemic anticoagulation was initiated after multidisciplinary discussion, balancing the risk of ongoing embolization against severe thrombocytopenia and active DIC. Despite antimicrobial therapy and anticoagulation, the patient’s hospital course was complicated by persistent anemia, progressive renal dysfunction, bilateral pleural effusions, and worsening peripheral gangrene. Rheumatologic evaluation was unrevealing, supporting a secondary process related to severe sepsis and coagulopathy.

Cardiothoracic surgery deemed the patient a prohibitive operative candidate due to active sepsis, coagulopathy, and multiorgan failure. Multidisciplinary discussions involving cardiology, infectious disease, critical care, cardiothoracic surgery, and interventional teams emphasized the high embolic and hemodynamic risk posed by the RA mass. Percutaneous aspiration using the AngioVac system (AngioDynamics, Latham, NY, USA) was therefore pursued, and the patient was transferred to a tertiary care center for advanced intervention. At the time of transfer, he remained critically ill but hemodynamically stabilized on reduced vasopressor support.

## Discussion

Mycotic RA masses present a rare but highly challenging clinical scenario, particularly in critically ill patients with multiorgan dysfunction. Right-sided cardiac masses pose a significant diagnostic and therapeutic challenge, with a differential diagnosis that includes thrombus, benign or malignant tumors, and IE [1,2]. RSIE accounts for approximately 5-10% of IE cases and is most commonly associated with intravenous drug use, indwelling vascular access, or intracardiac devices [1,3]. Although less common than left-sided IE, RSIE is associated with considerable morbidity due to embolic phenomena, severe tricuspid regurgitation, and right ventricular dysfunction. Large RA masses are particularly uncommon and often present with overlapping imaging characteristics, making definitive differentiation between vegetation and thrombus challenging, especially in critically ill patients [2].

In this case, multimodal imaging played a central role in risk stratification and clinical decision-making. Transthoracic echocardiography suggested a right-sided mass, and TEE revealed a 3 × 3 × 3 cm lobulated RA mass with a narrow pedunculated attachment prolapsing through the tricuspid valve into the right ventricle, resulting in severe tricuspid regurgitation. Imaging features favoring vegetation over thrombus included marked mobility, pedunculated attachment near the tricuspid annulus, and hemodynamic consequences on right ventricular function. CT pulmonary angiography additionally demonstrated a left segmental pulmonary embolism, consistent with a septic embolic phenomenon.

Microbiologic and clinical data supported a working diagnosis of RSIE. Blood cultures were positive for Streptococcus agalactiae, and targeted intravenous beta-lactam therapy was initiated. Critical care management included vasopressor support, careful fluid resuscitation, and cautious systemic anticoagulation. Rheumatologic evaluation was unrevealing, supporting a secondary process related to severe sepsis and coagulopathy.

Surgical intervention was deemed prohibitive due to active sepsis, coagulopathy, and multiorgan failure. In patients without these high-risk features, large vegetations, recurrent embolization, or severe valvular dysfunction are typical indications for surgical management. Minimally invasive percutaneous aspiration, such as the AngioVac system, has emerged as a viable alternative for high-risk surgical candidates, allowing for mass debulking and embolic risk mitigation [4]. This patient was successfully transferred to a higher-level care facility for AngioVac intervention.

This case highlights the diagnostic and management complexity inherent to large RA masses. Management strategies reported in the literature include targeted antimicrobial therapy, supportive critical care, systemic anticoagulation when indicated, surgical intervention for select patients, and minimally invasive percutaneous strategies for high-risk candidates. Early recognition, multimodal imaging, and multidisciplinary collaboration are essential for optimizing outcomes, particularly when histopathologic confirmation is unavailable and surgical risk is prohibitive.

## Conclusions

Large RA masses with hemodynamic consequences represent a significant diagnostic and therapeutic challenge. Multimodal cardiac imaging is essential for risk stratification and management planning, particularly when differentiation between thrombus and infective vegetation remains uncertain. In critically ill patients with prohibitive surgical risk, percutaneous aspiration may serve as a viable escalation strategy within a multidisciplinary care model. This case underscores the importance of early recognition, collaborative decision-making, and individualized escalation of care in complex presentations of RSIE.
